# A comment on participant reimbursement within Australian drug and alcohol research

**DOI:** 10.1111/dar.13452

**Published:** 2022-02-28

**Authors:** Daniel T. Winter, Brennan Geiger, Carolyn A. Day

**Affiliations:** ^1^ Specialty of Addiction Medicine, Central Clinical School, Faculty of Medicine and Health The University of Sydney Sydney Australia; ^2^ Edith Collins Centre (Translational Research in Alcohol Drugs and Toxicology) Sydney Local Health District Sydney Australia

Financial reimbursement for participation in drug and alcohol research has long been considered appropriate and is standard practice in Australia [1, 2]. Such payments afford a means to acknowledge participant value in advancing scientific knowledge, while recognising the time, inconvenience and expenses incurred. Furthermore, payments are considered scientifically and ethically valid [2, 3], can facilitate the recruitment of hidden or priority populations, and may increase retention in studies that require follow‐up [4, 5].

Financial reimbursements, cash or shopping vouchers, are commonly provided as methods of payment for research participation [6]. However, while vouchers provide an equivalent monetary value to cash, they often restrict the recipient's autonomy by limiting use at specific retailers or may restrict the purchase of certain goods (e.g. alcohol or tobacco products). Vouchers can be traded by participants for cash, often at a depreciated value, as has been noted by some researchers [7].

Cash, on the other hand, provides greater flexibility and autonomy for participants, and use toward non‐retail items, such as transport, personal grooming, rental or utility payments or pocket‐money for dependents. Cash, however, can pose real and important challenges for administering institutions, in terms of both financial administration and, potentially, staff security. Non‐financial reimbursements also exist (e.g. prize draws or raffles for vouchers) which, comparatively, provide little to no compensation for participation in research.

As we have previously described, there is currently little guidance around appropriate reimbursement practices for drug and alcohol research [8]. Most mechanisms for reimbursement are reliant upon individual researchers, availability of funds or institutional policies (which may be neither consumer nor evidence informed) to determine the type and value of reimbursement. As such, discrepancies in reimbursement provided across similar studies may exist or could be dictated solely by the sponsoring organisation without consideration of the broader ethical and scientific implications. Although there is broad guidance provided by the National Health and Medical Research Council on models for providing payments in research [3] and examples of payment policies for general consumer engagement, such as the National Mental Health Commission's Paid Participation Policy [9], there is no current policy on reimbursement in the drug and alcohol setting. Similarly, we are unaware of a scientific directive, such as the CONSORT (Consolidated Standards of Reporting Trials) [10] and STROBE (Strengthening the Reporting of Observational Studies in Epidemiology) [11] statements, to report whether reimbursement is provided for research and, if so, the type and value of the reimbursement. Given the scientific and ethical implications of participant reimbursements, this is cause for concern.

In examining reporting of reimbursements for research published in *Drug and Alcohol Review* from 2017 to 2019 (Volumes 36–38), we found 215 research papers involving individual participants who may use a substance. Of these, 43% of papers were from research based in Australia, with half (51%) providing at least some information regarding reimbursement within the paper, and a further 13% by referencing methodology elsewhere.

Of the Australian papers, 13% specified reimbursement with vouchers, 3% by cash/money transfer and 13% explicitly mentioned that no form of reimbursement was offered for participation. Non‐financial reimbursements were also identified (12%), most often a prize draw for a voucher or item of a large value. The remaining papers did not report on reimbursement. For those papers that did report reimbursement, however, one‐quarter did not report the payment mode method (i.e. if cash or voucher was provided) and 5% did not report value (e.g. ‘Participants received a gift voucher in recognition of their contribution’).

For those papers that did provide details, the value of the reimbursement ranged from $10 to 80 AU (mean = $36.6 AU, SD = 14.08) and 44% also reported the approximate duration to complete the study tasks (mean = 46.7 min, SD = 26.75; range = 5–150 min). Five papers reported varied reimbursement value based on factors such as age (i.e. lower reimbursement value for those underaged), location of study (i.e. varied, or no, reimbursement based on location of study) or driven by local consumer perceptions as to what is considered fair value for reimbursement.

Most papers examined issues related to general drug use (32%), alcohol, (23%), tobacco/nicotine (19%) and opioids (13%). Financial reimbursements were most often provided for opioid‐related research (75%), compared to alcohol (14%) and general drug use (37%) research. All other papers, based on substance type, provided financial reimbursement in 50% of cases.

Almost half (46%) of the papers assessed were focused on general population recruitment, specific populations recruited included people who use drugs (23%), people in or seeking drug and alcohol treatment (11%), and Aboriginal or Torres Strait Islander peoples (7%), among others. Financial reimbursements were commonly provided for inmates (100%), people who use drugs (77%), Aboriginal and Torres Strait Islander peoples (71%), but was less frequently provided in studies that recruited from the general population (21%) or those underaged/school students (17%).

Just over half (55%) of studies recruited from outside of health‐care services. Where recruitment occurred within or, in collaboration with, a health‐care service, just over half provided financial reimbursements (57%). Studies that did not involve a health service in recruitment reported the provision of reimbursement less often (27%).

We applaud the reported use of financial reimbursement in research, such as with people who use drugs and in opioid‐related research (77% and 75% of papers, respectively), which is consistent with both ethical and scientific principles described previously [1, 2, 3]. However, this raises two important questions. First, should these basic principles be extended to all drug and alcohol research, or indeed research in general? Second, is there an appropriate amount to provide for participation and, if so, what is that and how should this relate to participant's time spent on the research? We argue that the later point can only be answered in close consultation with relevant consumer groups. The former point regarding principles of reimbursement should at least be considered for all groups and also requires greater consumer input. However, this is arguably more challenging outside the illicit and, especially, the injecting drug use areas, or other areas of research where consumer representation may be less established.

We also argue for clear and precise reporting on participant reimbursement in all research papers. While it is evident that providing a financial reimbursement to participants is generally accepted in drug and alcohol research, particularly in Australia, there is arguably greater need for transparency, clarity and openness regarding disclosure of participant reimbursement within the literature to ensure ethical and scientific principles of research are upheld.

In conclusion, greater discussion and consideration for guidance regarding appropriate reimbursement is warranted across the drug and alcohol research sector, and consumers must play a central role in such a process. Without input from consumers, in determining whether participants should be reimbursed for research participation and the type and value of that reimbursement, researchers are inadvertently assessing the ‘value’ of the participant. Finally, research reports should be more transparent in their reporting of participant payment to increase scientific rigour and reproducibility.

## Conflict of Interest

The authors have no conflicts of interest.
